# Granulocyte colony stimulating factor therapy for stroke: A pairwise meta-analysis of randomized controlled trial

**DOI:** 10.1371/journal.pone.0175774

**Published:** 2017-04-13

**Authors:** Xin Huang, Yu Liu, Shuang Bai, Lidan Peng, Boai Zhang, Hong Lu

**Affiliations:** Department of Neurology, The First Affiliated Hospital of Zhengzhou University, Henan, China; Cardiff University, UNITED KINGDOM

## Abstract

Granulocyte colony-stimulating factor (G-CSF) is atherapeutic candidate for stroke that has demonstrated anti-inflammatory and neuroprotective properties. Data from preclinical and clinical studies have suggested the safety and efficacy of G-CSF in stroke; however, the exact effects and utility of this cytokine in patients remain disputed. We performed a meta-analysis of randomized controlled trials of G-CSF in ischemic and hemorrhagic stroke to assess its clinical safety and efficacy. Electronic databases were searched for relevant publications in English and Chinese. A total of 14 trials met the inclusion criteria. G-CSF (cumulative dose range, 1–135μg/kg/day) was tested against placebo in a total of 1037 participants. There was no difference in the rate of mortality between groups (odds ratio, 1.23; 95% confidence interval, 0.76–1.97, *p* = 0.40). Moreover, the rate of serious adverse events did not differ between groups and provided evidence for the safety of G-CSF administration in stroke patients (odds ratio, 1.11; 95% confidence interval, 0.77–1.61, *p* = 0.57). No significant outcome benefits were noted with respect to the National Institutes of Health Stroke Scale (mean difference, -0.16; 95% confidence interval, -1.02–0.70, *p* = 0.72); however, improvements were noted with respect to the Barthel Index (mean difference, 8.65; 95% confidence interval 0.98–16.32; *p* = 0.03). In conclusion, it appears to be safe in administration of G-CSF, but it will increase leukocyte count. G-CSF was weakly significant benefit with improving the BI scores, while there was no improvement in the NIHSS scores. Larger and more robustly designed trials of G-CSF in stroke are needed to confirm the results.

## Introduction

Stroke is a major cause of death and disability around the world that affects millions of patients every year. Additionally, the number of patients suffering from stroke is growing due to increasing aging populations. Thrombolysis is one of the few effective treatments for stroke; however, a narrow therapeutic window has limited its application in clinical practice. Other conventional pharmacotherapies for stroke including fibrinolytic, anticoagulant, and antiplatelet agents also have inadequate efficacy. Furthermore, while surgical treatments such as hemicraniectomy have been shown to increase the probability of survival among patients with malignant middle-cerebral-artery infarction, surgery carries the risk of producing substantial disability [1]. Therefore, better methods for the protection and recovery of damaged brain tissues after an established cerebral infarctionare required.

Granulocyte colony-stimulating factor (G-CSF) is a 20-kDa glycoprotein that belongs to the cytokine family of growth factors and functions to promote the production, mobilization, and differentiation of hematopoietic stem cells[2, 3]. G-CSF therapies including filgrastim, lenograstim, and pegfilgrastim have been approved for clinical use and utilized around the world for more than 20 years. G-CSF is widely used to promote the proliferation of granulocytes or APCs, for the treatment of congenital or acquired neutropenia, and for the mobilization of transplanted stem cells in patients with hematologic malignancies [4]. In addition to the functions of G-CSF in the hematopoietic system, recent trials have indicated that G-CSF may also play an important role in the central nervous system (CNS). Specifically, studies have suggested that G-CSF therapy can produce beneficial effects in stroke and improve neurological outcomes [5, 6]. In the CNS, G-CSF is thought to exert neuroprotective effects via the inhibition of apoptosis and inflammation [7] as well as by stimulating angiogenesis [8] and neurogenesis [9]. Indeed, an increasing number of patent applications are related to the development of G-CSF for the treatment of neurological disorders. Yet, the results of clinical trials of G-CSF agents in stroke are conflicting. Fan [10] had performed a meta-analysis that showed the improvement of functional outcomes in stroke patients treated with G-CSF in 2014. Their results were different to another review [11] performed by Bath in 2012. In the latest years, there had several new trials performed. To clarify the utility of G-CSF therapy after stroke, we conducted a meta-analysis of relevant clinical articles with the aim of informing the clinical efficacy of G-CSF in stroke and guiding future clinical studies.

## Materials and methods

### Search strategy

We conducted a search of the PubMed, Embase, Cochrane Library, Chinese Wanfang databases and SinoMed for relevant articles published between June of 1960 and February of 2016. Search terms included “stroke” or “cerebrovascular accident” and “G-CSF” or “granulocyte colony-stimulating factor.” Only articles published in English or Chinese were included. Additionally, a manual search was conducted on the bibliographies of relevant articles to identify eligible studies not referenced in the aforementioned databases.

### Selection of studies and data extraction

We only included original parallel-design randomized controlled trial studies that compared G-CSF therapy to placebo or no intervention in patients with acute or subacute ischemic or hemorrhagic stroke. All included trials reported mortality or severe adverse events (SAEs) as primary outcomes, and secondary outcomes in inclusion criteria included the National Institutes of Health Stroke Scale (NIHSS, a stroke severity scale) or Barthel Index (BI, scale for the activities of daily living and motor function assessment). Included trials also reported laboratory parameters for leukocyte counts. Studies were excluded if subjects did not meet the criteria for stroke as defined by the World Health Organization or if patients suffered from other major medical comorbidities (e.g., heart disease).

Each study was examined by 2 independent investigators in a 2-stage process as follows: first, titles and abstracts underwent initial screening; subsequently, the full-text articles were assessed for eligibility. Disagreements regarding inclusion were resolved through discussion with a third investigator. Dichotomous and continuous outcome measure data were extracted into data extraction tables by 2 independent investigators. Disagreements regarding the data were resolved through discussion with a third investigator. Trial authors were contacted for additional information when data reporting was insufficient or missing in original articles. Review Manager software version 5.3 was used for the analysis.

### Assessment of risk of bias

Risk of bias was assessed using the Cochrane Collaboration tool for assessment of risk of bias with the following items: (1) random sequence generation; (2) allocation concealment; (3) blinding of participants and personnel; (4) blinding of outcome assessment; (5) incomplete outcome data; (6) selective reporting; (7) other bias. Risk of bias was assigned as low, unclear, or high. Reporting bias was not assessed using funnel plots since the number of included trials was small.

### Assessment of heterogeneity

We tested for heterogeneity among studies using Q and *I*^2^ statistics. Heterogeneity was considered to be significant if the *p*-value of a given Q statistic was < 0.10. For *I*^2^ statistics, we classified heterogeneity as follows:*I*^2^ < 40% was classified as minimal; *I*^2^ = 40–75% was classified as modest; and *I*^2^ > 75% was classified as substantial [12]. Positive heterogeneity results were confirmed using a sensitivity analysis. For data synthesis, if statistical heterogeneity existed (*p* < 0.10 or *I*^2^ > 50%), we reported a random-effects model; if statistical heterogeneity did not exist (*p* > 0.10 or *I*^2^ < 50%), we reported a fixed-effect model.

### Statistics

The results of continuous outcomes were analyzed using mean difference (MD), with 95% confidence intervals (CI). The results of dichotomous outcomes were analyzed using odds ratios (OR) with 95% CI. We did not perform subgroup analyses since there were not sufficient trails with enough meaningful data.

## Results

### Search results

**Fig 1:**
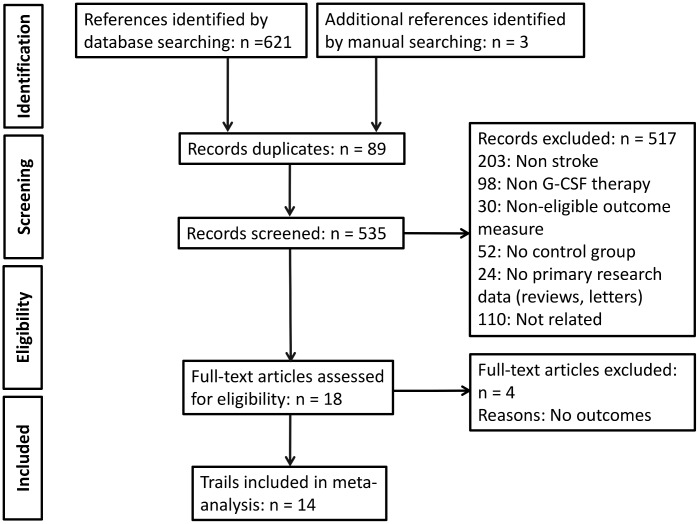
Study flow diagram.

A total of 621 articles were identified for review; 535 records remained after de-duplication. The initial screening excluded 517 studies, leaving a total of 18 references for full text assessment. Finally, 14articles met the inclusion criteria for our meta-analysis (Fig 1).

### Study characteristics

The characteristics of included studies are summarized in Table 1. A total of 1037 participants from 14 trials were included. Sample sizes ranged from 10 to 323 participants. The dosage of G-CSF administration varied according to each study, and the duration of treatment was 3, 5, or 7 days. Control groups were treated with placebo or conventional management. The length of follow up ranged from 14 days to 12 months. Included trials were conducted in China, Germany, Japan, Russia, India, and British.

**Table 1:** Characteristics of included studies.

Author and year of publication	Country	Subjects (Interv., Ctrl.)	Age (Interv., Ctrl.. years)	Method of administration	Dtime	Stroke type	Follow up period	Outcomemeasures
Ge 2005[13]	China	12; 13	__; __	Subcutaneous 5 μg/kg/d, 7d	<7 d	Acute ischemic stroke	6 mo	MESSS,BI
Li 2005[14]	China	36; 35	__; __	Subcutaneous 300 μg/d, 5d	Non-state	Acute stroke	3 mo	BI
Zhang 2006[15]	China	25; 25	60.5±5.9; 62.1±6.4	Subcutaneous 2 μg/kg/d, 5d	1 week	Acute stroke	20d	NIHSS
Sprigg 2006[16]	British	24; 12	76±9; 74±8	Subcutaneous 1, 3, or 10 μg/kg/d,1 or 5d	7–10 d	Ischemic stroke	3 mo	SNSS, BI, mRS
Shyu 2006[5]	Taiwan (China)	7; 3	64.0±10.5; 69.0±1.5	Subcutaneous 15 μg/kg/d, 5d	7 d	Acute ischemic stroke	12 mo	NIHSS, BI
Schäbitz 2010[17]	Germany	30; 14	71.1±11.4; 68.4±14.4	Intravenous 30,90, 135, or 180 μg/kg, 3 d	12 h	Acute ischemic stroke	90 d	NIHSS, BI, mRS
Xin 2011[18]	China	40; 40	55±10; 56±10	Intravenous 300 μg/d, 5d	<3 d	Acute stroke	14 d	BI
England 2011[19]	British	40; 20	71.1±12.9; 72.3±9.6	Subcutaneous 10 μg/kg/d,5d	3–30 d	Subacute stroke	3 mo	NIHSS
Alasheev 2011[20]	Russia	10; 10	50(46–57); 54(45–57)	Subcutaneous 10 μg/kg/d,5d	≤48 h	Acute ischemic stroke	3–6 mo	NIHSS, BI
Prasad 2011[21]	India	5; 5	__; __	Subcutaneous 10 μg/kg/d,5d	5 d	Acute ischemic stroke	6 mo	NIHSS, BI, mRS
Zhou 2013[22]	China	40; 42	63(9); 64.5(7)	Subcutaneous 600 μg/d,5d	<7 d	Acute stroke	3 mo	NIHSS, BI
Ringelstein 2013[23]	Germany	161; 163	69.3±0.9; 69.4±0.9	Intravenous 135μg/kg, 72h	≤9 h	Acute ischemic stroke	3 mo	mRS, NIHSS
Huang 2015[24]	China	50; 50	63.3±11.7; 62.2±12.8	Intravenous 300 μg/d, 5d	≤48 h	Acute ischemic stroke	14d	NIHSS, BI
Atsushi 2016[25]	Japan	39; 10	__; 71±13	Intravenous 150 or 300 μg/d, 5d	≤24 h	Acute ischemic stroke	3 mo	NIHSS

### Risk of bias in included studies

In summary, the overall risk of bias among the 14 studies was low (S1 and S2 Figs). Regarding the methods of randomization used, 12 studies were randomized using computer-generated random numbers, randomization tables, an interactive web response system, or a central randomization/allocation system; 2 studies were randomized using opaque envelopes. With regard to allocation, 7 trials provided adequate allocation concealment. For blinding, 5 studies were double-blinded and 1 study was open-label. In 9 studies, the blinding of outcome assessors was adequate. In 2 studies, 10% and 20% of participants were lost due to follow up, respectively. No selective reporting was identified, but it was also difficult to rule out the possibility of selective reporting in most of the trials. As for other bias, investigators in 2 trails held a patent using G-CSF for the treatment of stroke.

### Intervention effects

#### Mortality

**Fig 2:**
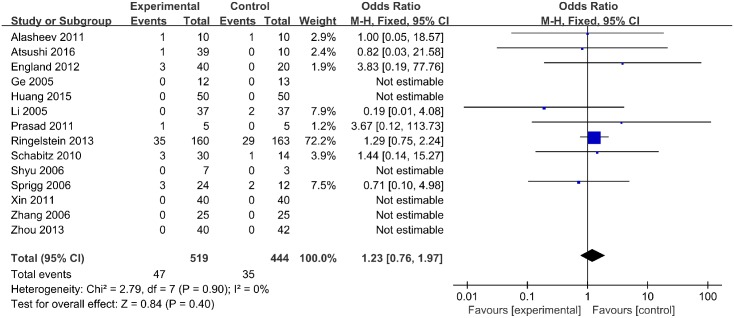
Mortality.

Mortality was reported for 519 treated subjects and 444 placebo subjects in 14 studies. In 6 studies, no deaths were reported. Overall, the rate of mortality did not differ between groups (odd ratio [OR], 1.23; 95% confidence interval [CI], 0.76–1.97; *p* = 0.40) (Fig 2). No statistical heterogeneity was observed.

#### SAEs

**Fig 3:**
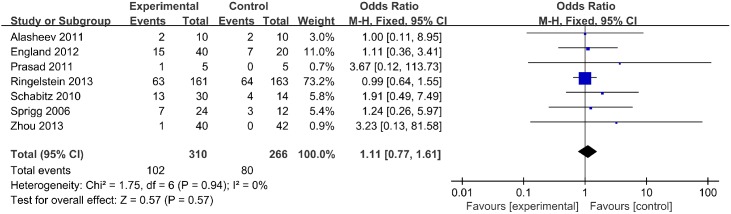
Severe adverse events.

SAEs were reported for 576 participants in 7studies. The incidence of SAEs was significantly different between groups (OR, 1.11; 95% CI, 0.77–1.61; *p* = 0.57) (Fig 3).

#### NIHSS scores

**Fig 4:**
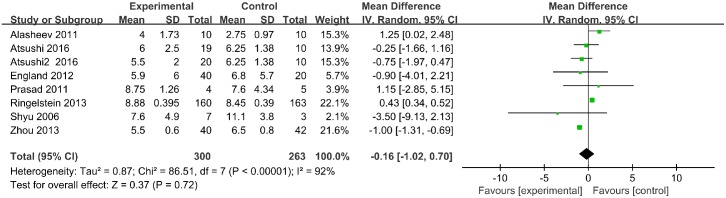
National Institutes of Health Stroke Scale scores.

NIHSS scores were reported over a follow up period exceeding 3 months for 563 participants in 7 trials. At 3 months (90 days) follow up, NIHSS scores were not significantly different between groups (mean difference [MD], -0.16; 95% CI, -1.02–0.70; *p* = 0.72) (Fig 4).

#### BI scores

**Fig 5:**
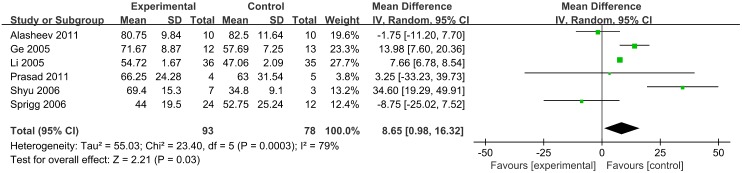
Barthel Index scores.

BI scores were reported over a follow up period exceeding 3 months for 171 participants in 6 trials. At 3 months (90 days) follow up, BI scores were lower in the G-CSF treatment group than the control group (MD, 8.65; 95% CI, 0.98–16.32; *p* = 0.03) (Fig 5).

#### Leukocyte counts

**Fig 6:**
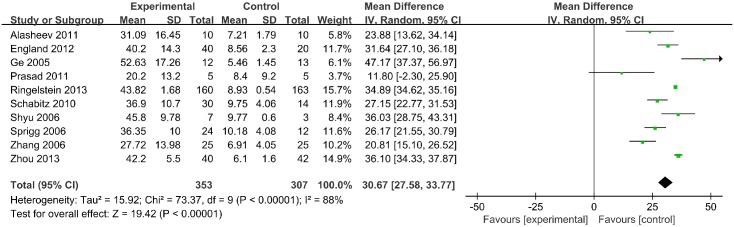
Leukocyte counts.

Leukocyte counts were reported for 660 patients in 10 trials. A significant increase in leukocyte count was identified in the G-CSF treatment group relative to the control group (MD, 30.67; 95% CI, 27.58–33.77; *p* <1 × 10^−5^) (Fig 6).

## Discussion

### Summary of main results

While some clinical trials have reported the feasibility, safety, and efficacy of G-CSF therapy for stroke [5], the exact effects and utility of G-CSF are still disputed. In the present meta-analysis, we retrospectively analyzed 14 trials of G-CSF therapy in stroke and did not identify adequate evidence for the beneficial effects of this treatment modality in patients. Specifically, no favorable effects were noted on stroke outcomes including NIHSS score, the incidence of SAEs, and mortality in patients treated with G-CSF versus control or placebo-treated patients. Interestingly, a beneficial effect of G-CSF therapy was observed on BI score; however, considering the small sample sizes of the included studies, this finding was insufficient to support the efficacy of G-CSF therapy in stroke.

Much work has been conducted to inform the potential mechanisms G-CSF in stroke. One potential mechanism is the immune modulatory actions of G-CSF [26, 27]. Administration of G-CSF in humans produces anti-inflammatory effects by promoting anti-inflammatory mediators (i.e., sTNF-R and IL-1ra) and decreasing the release of pro-inflammatory mediators (i.e., TNF, IFN-γ, and GM-CSF) [28]. Animal studies have confirmed the ability of G-CSF to decrease the release of pro-inflammatory cytokines (TNF, IFN-γ and IL-6) and enhance the production of anti-inflammatory cytokines such as IL-4 [29]. Furthermore, G-CSF suppresses the inflammatory response of monocytes/macrophages (TNF, IL-8, IL-12) to toll-like receptor activation *in vitro* [26, 30–32]. Though several reviews have addressed the ability of G-CSF to reduce infarct volume and improve functional outcomes in animal models of stroke [33, 34], it has been difficult to translate these results to the clinical setting. G-CSF may have different neuroprotective efficacy in different phases and/or subtypes of stroke. For example, studies in acute ischemic stroke have shown greater improvements in neurologic function after more than 3 months of follow up studies [5, 13]. Future studies should investigate the utility of G-CSF in sub-acute versus chronic stroke, and in ischemic versus hemorrhagic stroke.

### Quality of evidence

The total number of participants included in the present meta-analysis was relatively small and thus inadequate for making definitive conclusions about the safety and efficacy of G-CSF therapy in stroke. Moreover, the follow up duration was less than 12 months in all studies and therefore provided an incomplete assessment of the safety of G-CSF therapy. The onset of G-CSF administration varied from 9 hours to 30 days after stroke onset, which may also have obscured the efficacy of G-CSF therapy. Three trails showed performance bias, and 5 trails did not report details about the blinding of participants and personnel. Though the overall data had a low risk of bias, the results of the current meta-analysis should be interpreted with caution.

### Literature context

At the time of this study, we identified 2 other meta-analyses that assessed the efficacy of G-CSF therapy in patients with stroke [10, 11]. The review by Fan included 10 studies with 711 patients and the other one included 8 trials involving 548 participants. Both reviews showed that G-CSF produced minor side effects but was safe overall, and indicated that G-CSF significantly increased leukocyte counts; these findings were similar to the results of our analysis. With regard to functional outcomes, the review by Fan et al. found significant improvements in NIHSS score in the G-CSF group versus the placebo group (P< 0.05), whereas the review by Bath et al. only detected a non-significant reduction in early functional impairment. With regard to NIHSS score, our study was in agreement with the latter report. However, the observed effect of G-CSF therapy on BI score in our study represents a novel finding. Considering that reviews with varied sample size had shown different results, we suggest that larger and more robustly designed trials of G-CSF in stroke are needed in the future.

## Conclusions

Evidence from 14 studies that met the inclusion criteria for this meta-analysis indicated that G-CSF treatment in a cumulative dosage range from1–135 μg/kg/day did not produce significant SAEs in stroke patients; however, the small numbers of participants included in each trial made it difficult to make definitive conclusions about the safety of G-CSF therapy in stroke patients, especially considering the observed effect on leukocyte count. Moreover, our results showed no effect of G-CSF therapy on NIHSS score or other outcomes but a positive effect on BI score relative to control. Larger RCTs are needed to evaluate the efficacy of G-CSF in stroke.

## Supporting information

S1 Fig Risk of bias graph. (TIF)

S2 Fig Risk of bias summary. (TIF)

S1 File Search strategies. (DOC)

S2 File PRISMA 2009 checklist. (DOC)
