# Supplementary figures and images for: Granulocyte colony stimulating factor therapy for stroke: A pairwise meta-analysis of randomized controlled trial
Source: PLoS One. 2017 Apr 13;12(4):e0175774. doi: 10.1371/journal.pone.0175774 (PMC5391086; doi:10.1371/journal.pone.0175774)

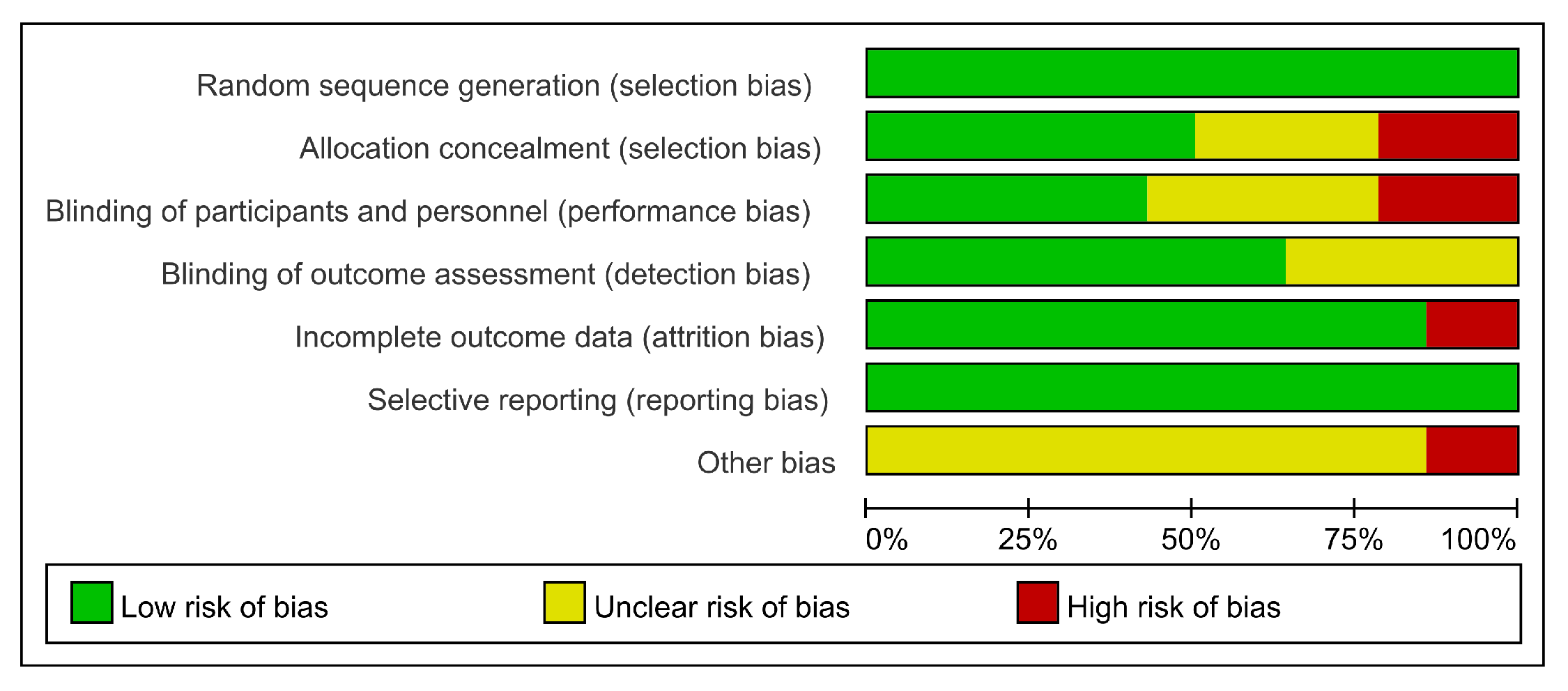

Supplement: S1 Fig — (TIF) [file pone.0175774.s001.tif]

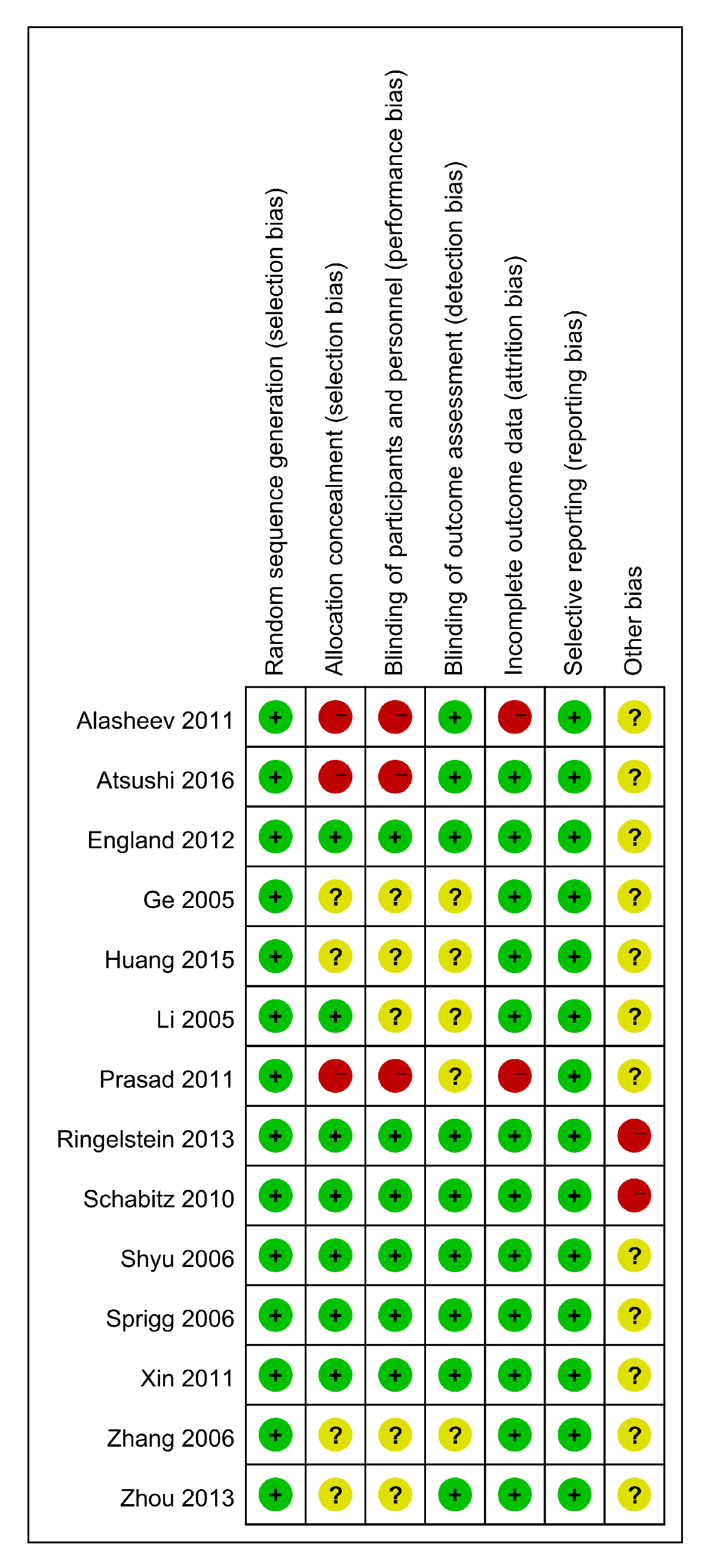

Supplement: S2 Fig — (TIF) [file pone.0175774.s002.tif]
