# Supplementary material for: Granulocyte colony stimulating factor therapy for stroke: A pairwise meta-analysis of randomized controlled trial
Source: PLoS One. 2017 Apr 13;12(4):e0175774. doi: 10.1371/journal.pone.0175774 (PMC5391086; doi:10.1371/journal.pone.0175774)
Supplement: S1 File — (DOC) [file pone.0175774.s003.doc]

**Search strategies**

**Pubmed**

#1 “stroke”[Mesh]

#2 “stroke*”[Text Word]

#3 “Cerebrovascular Accident*” [Text Word]

#4 “CVA*” [Text Word]

#5 “Cerebrovascular Apoplexy” [Text Word]

#6 “Brain Vascular Accident*” [Text Word]

#7 “Cerebrovascular Stroke*” [Text Word]

#8 “Apoplexy” [Text Word]

#9 “Cerebral Stroke*” [Text Word]

#10 “Acute Stroke*” [Text Word]

#11 “Acute Cerebrovascular Accident*” [Text Word]

#12 or/1-11

#13 “Granulocyte Colony-Stimulating Factor”[Mesh]

#14 “G-CSF” [Text Word]

#15 “colony-stimulating factor*” [Text Word]

#16 “filgrastim” [Text Word]

#17 “pegfilgrastim” [Text Word]

#18 “neupogen” [Text Word]

#19 “lenograstim” [Text Word]

#20 “molgramostim” [Text Word]

#21 or/13-20

#22 12 and 21

#23 “randomized controlled trial”[Publication Type]

#24 “controlled clinical trial”[Publication Type]

#25 “randomized” [Title/Abstract]

#26 “placebo” [Title/Abstract]

#27 “ randomly” [Title/Abstract]

#28 “trial” [Title/Abstract]

#29 “groups” [Title/Abstract]

#30 or/23-29

#31 22 and 30

Filters activated: Humans, English, Chinese.

**Embase**

#1 ‘cerebrovascular accident’/exp

#2 ‘cerebrovascular accident*’:ti,ab

#3 ‘stroke*’:ti,ab

#4 ‘CVA*’:ti,ab

#5 ‘cerebrovascular apoplexy’:ti,ab

#6 ‘apoplexy, cerebrovascular’:ti,ab

#7 ‘vascular accident*,brain’:ti,ab

#8 ‘brain vascular accident*’:ti,ab

#9 ‘cerebrovascular stroke*’:ti,ab

#10 ‘stroke*,cerebrovascular’:ti,ab

#11 ‘apoplexy’:ti,ab

#12 ‘cerebral stroke*’:ti,ab

#13 ‘stroke*,cerebral’:ti,ab

#14 ‘stroke*,acute’:ti,ab

#15 ‘acute stroke*’:ti,ab

#16 ‘cerebrovascular accident*,acute’:ti,ab

#17 ‘acute cerebrovascular accident*’:ti,ab

#18 or/1-17

#19 ‘granulocyte colony stimulating factor’/exp

#20 ‘g-csf’:ti,ab

#21 ‘colony-stimulating factor*’:ti,ab

#22 ‘filgrastim’:ti,ab

#23 ‘pegfilgrastim’:ti,ab

#24 ‘neupogen’:ti,ab

#25 ‘lenograstim’:ti,ab

#26 ‘molgramostim’:ti,ab

#27 or/19-26

#28 ‘randomized controlled trial’/exp

#29 ‘controlled clinical trial’:ti,ab

#30 ‘randomized’:ti,ab

#31 ‘placebo’:ti,ab

#32 ‘randomly’:ti,ab

#33 ‘trial’:ti,ab

#34 ‘groups’:ti,ab

#35 or/28-34

#36 #18 and #27 and #35（[chinese]/lim OR [english]/lim）AND [humans]/lim

**Cochrane Library**

#1 MeSH descriptor: [Stroke] explode all trees

#2 stroke*:ti,ab,kw

#3 cerebrovascular accident*:ti,ab,kw

#4 CVA*:ti,ab,kw

#5 cerebrovascular apoplexy:ti,ab,kw

#6 apoplexy, cerebrovascular:ti,ab,kw

#7 vascular accident*,brain:ti,ab,kw

#8 brain vascular accident*:ti,ab,kw

#9 cerebrovascular stroke*:ti,ab,kw

#10 stroke*,cerebrovascular:ti,ab,kw

#11 apoplexy:ti,ab,kw

#12 cerebral stroke*:ti,ab,kw

#13 stroke*,cerebral:ti,ab,kw

#14 stroke*,acute:ti,ab,kw

#15 acute stroke*:ti,ab,kw

#16 cerebrovascular accident*,acute:ti,ab,kw

#17 acute cerebrovascular accident*:ti,ab,kw

#18 or/1-17

#19 MeSH descriptor: [Granulocyte Colony-Stimulating Factor] explode all trees

#20 G-CSF:ti,ab,kw

#21 colony-stimulating factor*:ti,ab,kw

#22 filgrastim:ti,ab,kw

#23 pegfilgrastim:ti,ab,kw

#24 neupogen:ti,ab,kw

#25 lenograstim:ti,ab,kw

#26 molgramostim:ti,ab,kw

#27 or/19-26

#28 #18 and #27

**Chinese Wanfang Databases and SinoMed**

检索表达式：（主题：（卒中）or 题名或关键词：（中风）or题名或关键词：（脑梗死） or题名或关键词：（脑血管意外））and （主题：（集落刺激因子）or题名或关键词：（粒细胞集落刺激因子）or题名或关键词：（巨噬细胞集落刺激因子）or题名或关键词：（粒细胞巨噬细胞集落刺激因子）or题名或关键词：（白介素3）or题名或关键词：（重组集落刺激因子））
